# Modular titanium alloy neck adapter failures in hip replacement - failure mode analysis and influence of implant material

**DOI:** 10.1186/1471-2474-11-3

**Published:** 2010-01-04

**Authors:** Thomas M Grupp, Thomas Weik, Wilhelm Bloemer, Hanns-Peter Knaebel

**Affiliations:** 1 Aesculap AG Research & Development,Tuttlingen, Germany; 2 Aesculap AG Clinical Science,Tuttlingen, Germany

## Abstract

**Background:**

Modular neck adapters for hip arthroplasty stems allow the surgeon to modify CCD angle, offset and femoral anteversion intraoperatively. Fretting or crevice corrosion may lead to failure of such a modular device due to high loads or surface contamination inside the modular coupling. Unfortunately we have experienced such a failure of implants and now report our clinical experience with the failures in order to advance orthopaedic material research and joint replacement surgery.

The failed neck adapters were implanted between August 2004 and November 2006 a total of about 5000 devices. After this period, the titanium neck adapters were replaced by adapters out of cobalt-chromium. Until the end of 2008 in total 1.4% (n = 68) of the implanted titanium alloy neck adapters failed with an average time of 2.0 years (0.7 to 4.0 years) postoperatively. All, but one, patients were male, their average age being 57.4 years (36 to 75 years) and the average weight 102.3 kg (75 to 130 kg). The failures of neck adapters were divided into 66% with small CCD of 130° and 60% with head lengths of L or larger. Assuming an average time to failure of 2.8 years, the cumulative failure rate was calculated with 2.4%.

**Methods:**

A series of adapter failures of titanium alloy modular neck adapters in combination with a titanium alloy modular short hip stem was investigated. For patients having received this particular implant combination risk factors were identified which were associated with the occurence of implant failure. A Kaplan-Meier survival-failure-analysis was conducted. The retrieved implants were analysed using microscopic and chemical methods. Modes of failure were simulated in biomechanical tests. Comparative tests included modular neck adapters made of titanium alloy and cobalt chrome alloy material.

**Results:**

Retrieval examinations and biomechanical simulation revealed that primary micromotions initiated fretting within the modular tapered neck connection. A continuous abrasion and repassivation process with a subsequent cold welding at the titanium alloy modular interface. Surface layers of 10 - 30 μm titanium oxide were observed. Surface cracks caused by fretting or fretting corrosion finally lead to fatigue fracture of the titanium alloy modular neck adapters. Neck adapters made of cobalt chrome alloy show significantly reduced micromotions especially in case of contaminated cone connection. With a cobalt-chromium neck the micromotions can be reduced by a factor of 3 compared to the titanium neck. The incidence of fretting corrosion was also substantially lower with the cobalt-chromium neck configuration.

**Conclusions:**

Failure of modular titanium alloy neck adapters can be initiated by surface micromotions due to surface contamination or highly loaded implant components. In the present study, the patients at risk were men with an average weight over 100 kg. Modular cobalt chrome neck adapters provide higher safety compared to titanium alloy material.

## Background

Total Hip Arthroplasty (THA) has become a successful clinical treatment to restore the function of the joint, with a positive impact on the patient's quality of life. Modular connections for hip prostheses have been used since the early 70ies for heads with different neck sizes or diameters. Later in the 90ies, modular neck adapters have been introduced [1,2] for intraoperative adjustment of collum-caput-diaphysis (CCD) angle and femoral anteversion to optimise offset and leg length, irrespective of the hip stem implanted. These solutions have been proven their relevance in total hip arthroplasty [3,4]. Several failures of modular connections in hip replacement including primary and revision stem components [5-8] were reported. Long-term experiences with modular heads made of cobalt-chromium alloy (CoCr29Mo6) in combination with the cone of the stem out of titanium alloy (TiAl6V4) have revealed, apart from traces of fretting corrosion, no adverse events such as fractures of the tapers in clinical use [9-17]. Failures of modular neck adapters have been rarely documented [5-7]. In 2007, we reported three cases of a failure of a modular short hip stem [8]. The purpose of this paper is to present the state-of-the-art research and the recent findings of the failure analysis.

## Case history

**Figure 1:**
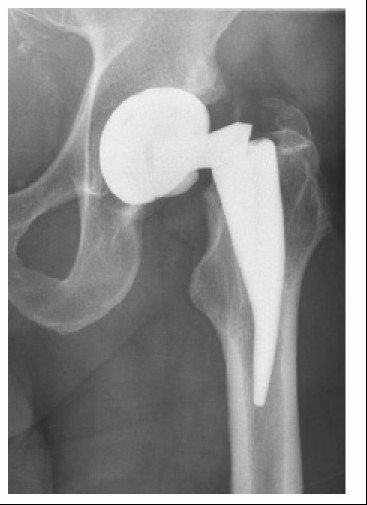
**X-ray of a hip with a failed neck**.

The failed neck adapters were implanted between August 2004 and November 2006. After the third incident, the titanium neck adapters were replaced by adapters out of cobalt-chromium [8]. A typical x-ray of a failed titanium neck adapter is shown in Figure 1.

**Figure 2:**
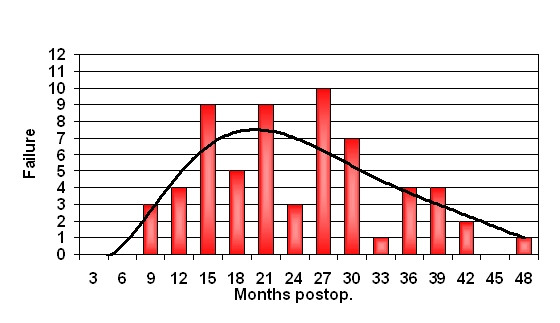
**Occurrence of clinical neck failures in a postoperative time period in months**.

**Figure 3:**
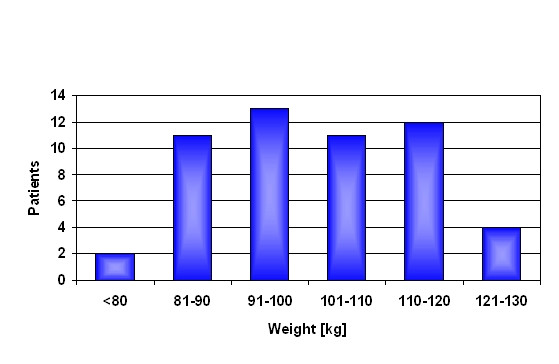
**Correlation weight of patients and failures**.

The failure of the neck did not cause further damage on the acetabulum side. Subsequently, there was no need to revise the cups in all of these cases. Modular cup inserts were replaced in some cases due to visible signs of damage originated by the neck failure or for reasons of precaution. About 5000 hip joints have been implanted with the titanium stem and titanium neck adapter material combination. Until the end of 2008 1.4% (n = 68) of the titanium alloy neck adapters failed after an average time in vivo of 24 months (Figure 2). All, but one, patients were male and most patients (59%) had a weight above 100 kg and an average body mass index (BMI) of 31.6 (24 to 42) (Figure 3).

An overview about the average age, weight and postoperative time until failure of the titanium alloy neck adapter in 68 patients and the distribution of neck adapter geometry, head size and length is given in Table 1. The implant size did not have a detectable influence on the occurrence of neck failure. The neck adapter failures were divided into 66% with a CCD angle of 130°, 34% with a CCD angle 135° and none with 140°.

**Table 1:** Overview about the average age, weight and time in vivo until failure of the titanium alloy neck adapter in 68 patients and distribution of neck adapter and combined head geometry

age	Ø 57.2 years (36 - 75 years)					
**weight**	Ø 102.3 kg (75 to 130 kg)					

**time in vivo**	Ø 24 months (8 to 48 months)					

**neck adapter** **(CCD-angle)**	130°/0°	130° ± 7.5°	135°/0°	135° ± 7.5°	140°/0°	140° ± 7.5°
	
	29	16	18	5	-	-

**head length**	S	M	L	XL	XXL	unknown
	
	4	22	26	7	6	3

**head diameter**	28 mm		32 mm		36 mm	
	
	11		50		7	

**head material**	CoCrMo		Biolox forte		Biolox delta	
	
	13		53		2	

**Figure 4:**
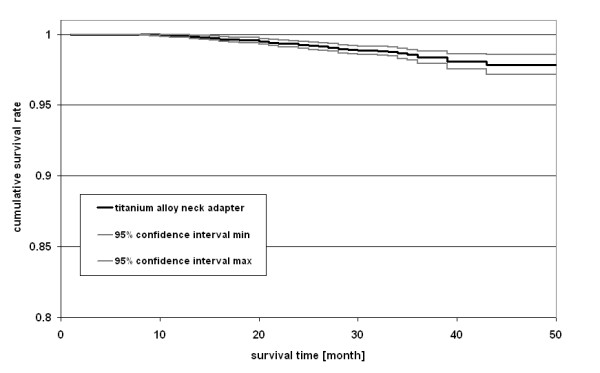
**Kaplan-Meier survival analysis for failed neck adapter as reason of revision (± 95% confidence interval)**.

A Kaplan-Meier survival analysis (Statistica 7, StatSoft Europe GmbH, Hamburg, Germany) was undertaken with revision due to failed neck adapter as an endpoint (confidence interval ± 95%). A cumulative survival rate of 98.6% was calculated with fractured neck adapter as the causative factor of failure (Figure 4).

There was no correlation between implant failures and a specific clinic or surgeon.

## Methods

### Implant component description

**Figure 5:**
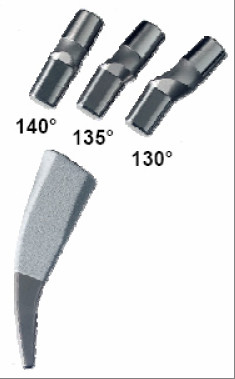
**Metha Short Hip Stem System**.

The modular neck adapters and stems used in our study were in all cases the Metha Short Hip Stem Prosthesis (Aesculap AG, Tuttlingen, Germany). Adapters and stems were made of titanium alloy. With its circumferencial pure titanium porous coating and additive thin layer of dicalcium phosphate dehydrate (Plasmapore^® ^μCaP, Aesculap, Tuttlingen, Germany), the stem is designed for cementless anchorage (Figure 5). The modular neck adapters were used in three different CCD-angles (130°, 135° and 140°) in combination with neutral, anteverted and retroverted (± 7.5°) versions to adjust the CCD-angle intraoperatively [4].

### Retrieval analysis

In the retrieval analysis, the failed neck adapters were subjected to a detailed examination to which most patients consented. The analysis is in every case based on the failed neck adapter and the hip stem including the remaining distal part of the neck adapter. The revised modular heads and cup inserts were in most of the cases not available and not subject of this examination. The present report is based on the retrieval analysis of 47 devices, the complete investigation data being compiled at the end of 2008. The fracture and modular connection surfaces were evaluated using light microscope and scanning electron microscope (SEM) (Zeiss EVO 50, Carl Zeiss NTS GmbH Oberkochen, Germany).

The fragment of the broken adapters which remained inside the shaft was cut through axially. One half of the adapters was cautiously removed from the shaft to examine the contact zone between the two components. The other half was left inside the shaft to enable the metallographic investigation of the interface and the microstructure of the materials. To enable the metallographic investigation of the neck adapter/stem interface the samples were prepared by mechanical cutting using a cut off-wheel (Secotom 10 Struers A/S, Denmark). After embedding into epoxy resin (Polyfast Struers A/S, Denmark) the cross section was prepared by grinding, polishing and etching according to the method by Kroll (composition 100 ml distilled H_2_O, 1 ml HF, 3 ml HNO_3_). The investigation was performed by light microscope (Wilde M3Z Herrenbrugg, Switzerland) with a magnification up to 500-times.

The chemical composition of the abrasive substances found in the crevice of the cone connection was determined using energy dispersive analysis (EDX) (Oxforth EDX-Analysensystem INCA 7059 Oberkochen, Germany) and wet chemical digestion. The surface of the cone was rinsed cautiously using dilute acid. After hot digestion and dilution the analysis of the elements was performed using inductively coupled plasma optical emission spectrometry (ICP-OES Horiba Ultima II Jobin Yvon Longjumeau, France).

### Micromotion analysis of the modular neck stem interface

**Figure 6:**
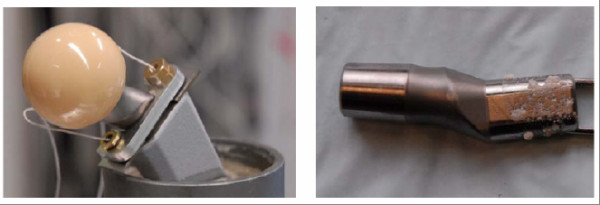
**Test setup for measurement of micromotions in the modular cone connection (left) and particle-contaminated joining area (right) **[18,19].

To quantify the relative motion in the interface of the modular hip stem out of titanium alloy a total of eight specimens with neck adapters out of titanium alloy and cobalt-chromium alloy were examined. As it appears that intraoperative contamination of the cone connection with bone particles has an considerable impact on the magnitude of fretting in the interface due to increased micromotions, a specific test setup was developed at the Biomechanics Section of the Hamburg University of Technology (Prof. Michael M. Morlock) and each device being tested with a clean and with a particle contaminated joining area (small porcine bone grafts approximately 1 mm in diameter) [18,19]. To determine the relative motion between neck adapter and stem a contactless measurement system (Micro-Epsilon Type U05(78) Messtechnik Ortenburg, Germany) was used. Two sensors with a range of 500 μm and a sensitivity of 0.025 μm were fixed on an aluminium plate and mounted on the neck adapter. Two steel plates fixed on the resection plane of the stem served as targets for the sensors (Figure 6).

The stem was combined with neck adapters with 130° CCD angle, embedded in bone cement (Palacos R, Heraeus Medical GmbH Wehrheim, Germany) and tested on a servohydraulic testing machine (MTS 850.2, MTS Systems Corporation Eden Prairie MN, USA). A sinusoidal axial force between 50 and 2500 N was applied via a ceramic head with neck length L at a frequency of 1 Hz for 2000 cycles to measure the relative displacement between neck adapter and stem with regard to irreversible settling and micromotions. A statistical analysis was performed to distinguish between independent groups (clean and particle contaminated joining area) (paired Student's t test) for both neck adapter materials (SPSS 15.0).

### Pre-clinical fatigue testing

To determine the endurance properties of the neck region comparative tests were performed according to ASTM F 2068-03 and ISO 7206-6:1992(E) (MTS Mini Bionix II, MTS Systems GmbH Berlin, Germany). The hip contact force was set at 5340 N with a sinusoidal loading mode (ratio F_min_/F_max _= 0.1). In a saline medium (0.9%, pH 7) the number of cycles was set at 10 million load alterations at a frequency of 15 Hz. In a worst case scenario using a 32 mm diameter XL head, neck fatigue was tested on the smallest modular stem in combination with neck components with 130° CCD angle and 7.5° retrotorsion out of titanium and cobalt-chromium alloy, respectively. A paired Student's t test was used to differentiate the fatigue behaviour of the two neck adapter materials (Statistica 7, StatSoft Europe GmbH, Hamburg, Germany).

**Figure 7:**
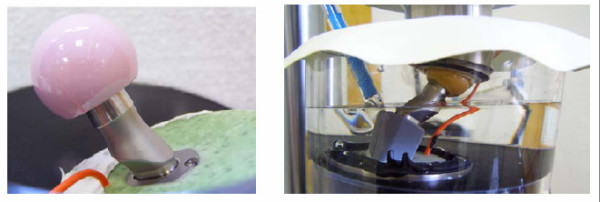
**Test setup for neck test (left) and stem test (right) with reference electrode to measure corrosion potential**.

To evaluate the coaction of the stem/neck modularity with the hip stem, the modular stems were tilted both in the frontal plane (α = 10°) and in the sagittal plane (β = 9°). Based on CT scans on several human femurs and the specific stem design the level of embedding was set to 45 mm below the centre of the head to simulate a deficiency of proximal support due to bone loss in the neck and trochanter region (Figure 7).

For these customised test, the hip contact force was set at 2300 N for 5 million cycles according ISO 7206-8:1995(E) followed by a stepwise load increase (Locati method 500 N, 1 million cycles) until failure. These customised test setup was introduced to analyse if the mode of fatigue failure occurs in the modularity or in the stem region outside of the neck/stem connection.

To simulate the clinical failure modes in vitro, parameters for the titanium neck adapters were varied as shown in Table 2.

**Table 2:** In vitro model to simulate the clinical failure modes with various parameters

Hip contact force	3800 N
**Frequency**	1 Hz/15 Hz

**Stress and rest phases**	1,000 cycles, 1.5 minutes rest, 1,000 cycles, 1.5 minutes rest repeat

**Contamination**	Dry, Blood, Serum, Cortical bone particles

**Surrounding medium**	Saline 0.9% pH 7 Saline 1.8% pH 2 CaCl_2 _solution pH 2 FeCl_3 _solution pH 0.5

The resultant hip contact force was set at 3800 N to simulate a higher strain as in overweight or active patients. The test frequency was decreased from 15 Hz to 1 Hz to detect a possible influence on fretting corrosion in the modular neck interface. To simulate the patient's situation in daily life more realistically frequent stress phases alternated with rest phases. Taking into account the clinical conditions of hip replacement assembly, the connection was contaminated with blood, serum and cortical bone particles (approximately 1 mm in diameter) to provoke a mechanical disturbance in the interface. Additionally, the surrounding chemical conditions were altered by modifying pH values and enhancing chloride concentrations with the use of NaCl, CaCl_2 _and FeCl_3 _to accelerate any eventual tribochemical processes. To determine the continuous abrasion and repassivation of the titanium alloy surfaces in the neck/stem connection the redox potential was measured (InLab^® ^301 Mettler Toledo Balingen, Germany).

## Results

### Findings of the retrieval examination in clinical failures

**Figure 8:**
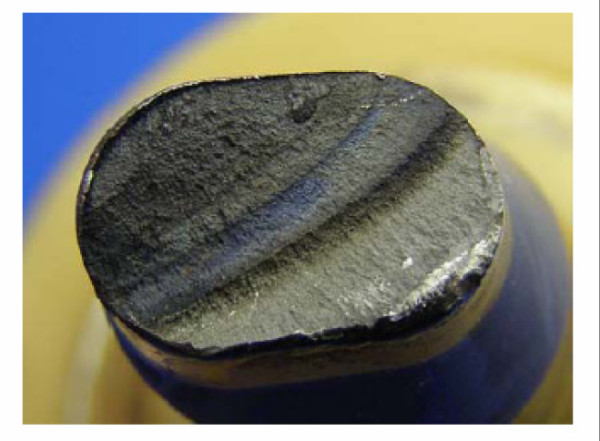
**Fatigue fracture surface of a clinically failed titanium neck adapter**.

All retrieved modular adapters showed similar breakage cross-sections. The fatigue fracture starts in the antero-lateral area at the upper part of the conical connection, the area subjected to maximum biomechanical stress. The fatigue fracture is followed by the residual forced fracture (Figure 8).

**Figure 9:**
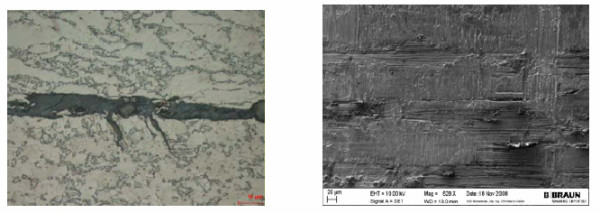
**Metallographic analyses revealed microcracks on the cone surface (left) and a potential microcrack in a fretting area (right)**.

Metallographic analyses showed that microcracks developed on the surface of the cone in the clamping range (Figure 9). It appears that these microcracks induced the fatigue crack which finally led to the implant failure. Microcracks were analyzed using a scanning electron microscope. Figure 9 shows a potential micro crack in an area where fretting marks can be seen on the surface of the cone.

**Figure 10:**
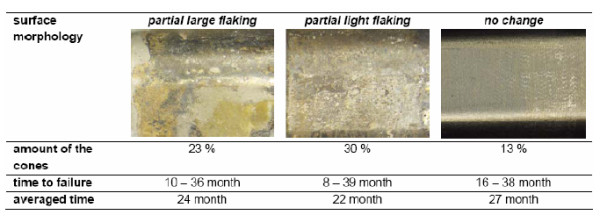
**Overview of interface characteristics and time to failure of the retrievals**.

The examination did not indicate any product deviation, manufacturing failure or batch correlation, thus excluding any manufacturing influence as cause for implant breakage. In the majority of cases, the surface of the cones was significantly modified and showed signs of fretting corrosion. Among the forty-seven of the revised cones available for examination, a total of 87% presented substantial fretting overlaid by laminar corrosion of different intensity (Figure 10).

**Figure 11:**
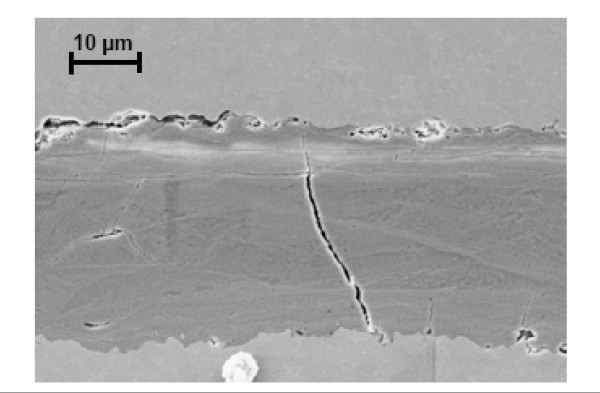
**Brittle layer in cone connection between stem and neck adapter**.

The findings of the corrosive alteration apart of fretting have not been corroborated by biomechanical tests so far [8,14]. Metallographic cross sections of the cone show a 20 μm up to 30 μm thick layer developing in the modular joining area of most of the revised implants (Figure 11), a layer composed of different abrasive and corrosive substances including a high amount of titanium dioxide. The layer was formed due to abrasion of the passive oxide layer protecting the titanium alloy against corrosion. Continued abrasion and repeated repassivation of the titanium alloy surface produced the oxide layer's characteristic thickness.

Additionally in some cases this process accelerated through crevice corrosion as the reduction of the pH to values of about 2-3 indicates.

**Figure 12:**
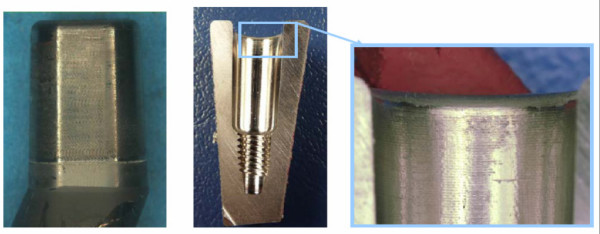
**Retrieved faultless titanium alloy neck adapter (left) and stem (right) without contamination and any signs of corrosion**.

In one patient weighing 100 kg, a revision had to be carried out after 26 months to improve the offset due to cup loosening, thus providing the possibility to examine a faultless titanium adapter. (Figure 12).

The surface of the cone adapter which was not cleaned after revision was examined using a scanning electron microscope. No calcium or phosphor could be detected. There was no indication of a contamination of the cone connection by bone particles. Besides mild abrasion on the surface, no signs of fretting corrosion could be found.

### Influence of micromotions on the modular neck stem interface

**Figure 13:**
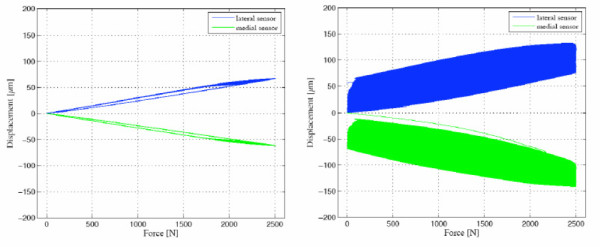
**Settling behaviour of a clean titanium alloy neck adapter (left) and a particle-contaminated joining (right) **[18].

The major part of the observed displacement was caused by elastic deformation of the neck adapter. The micromotions of the titanium neck adapter were in a clean condition 6 ± 4 μm (medial) and 6 ± 2 μm (lateral) and with particle contamination 18 ± 3 μm (medial) and 15 ± 4 μm (lateral). In each contaminated joining area, the displacement and the micromotions were larger than in a clean condition. The fully uncontaminated components showed a distinct and definitive settling within the first 20 cycles. In case of a contaminated joining area the settling process of the neck adapter did not come to an end and lasted over the entire test duration (2000 cycles) (Figure 13).

**Figure 14:**
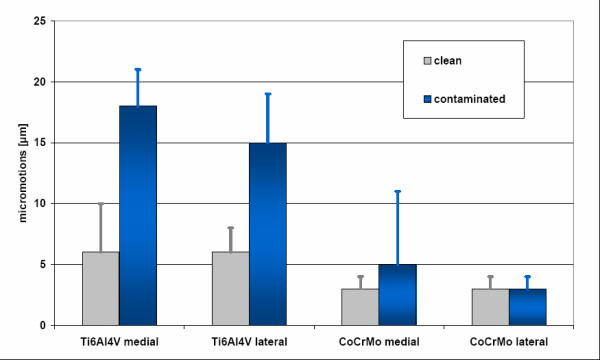
**Micromotions in a clean and particle-contaminated interface (Mean ± STD) for titanium and cobalt-chromium alloy neck adapters (medial and lateral)**.

The titanium alloy neck adapter showed a significant increase of micromotions in the interface of the particle-contaminated joining (p < 0.01) in comparison to clean conditions (Figure 14). The micromotions of the neck adapter out of cobalt-chromium were in a clean condition 3 ± 1 μm (medial) and 3 ± 1 μm (lateral) and with particle contamination 5 ± 6 μm (medial) and 3 ± 2 μm (lateral).

In the case of the cobalt-chromium alloy neck adapter, the contamination of the joining area did not influence the micromotions significantly (p = 0.43).

### In vitro fatigue behaviour of the stem/neck modularity

The neck adapters made of titanium and of cobalt-chromium alloy showed significantly different (p = 0.009) endurance behaviour. The Ti6Al4V adapters combined with a 28 L Biolox forte head failed after 2.45 million cycles (range 0.189 to 4.43). No failure occurred with the cobalt-chromium adapters (32 XL Biolox option) up to 20 million cycles.

**Figure 15:**
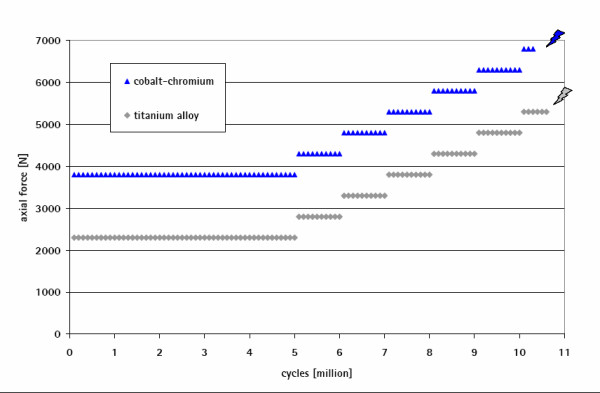
**Endurance behaviour of the modular stem and different failure mechanisms for neck components made of titanium (failure of the neck adapter) and of cobalt-chromium alloy (fractured stem at a level below the embedding)**.

Simulating a deficiency in proximal stem support the fatigue behaviour of the coaction of the stem/neck modularity with the hip stem was examined in a customised test setup. The titanium alloy neck adapters fulfilled the ISO 7206-8: 1995(E) requirements (2300 N for 5 million cycles) and failed during a stepwise increase of the applied force (Locati method) at a high load level of 5300 N after 0.7 million cycles (range 0.63 to 0.75). In a direct comparison, the cobalt-chromium neck adapters did not fail in the Locati test until the stem fractured at 6800 N load after 0.3 million cycles (Figure 15). Furthermore, the cobalt-chromium neck components did not fracture at 5300 N during 10 million cycles.

**Figure 16:**
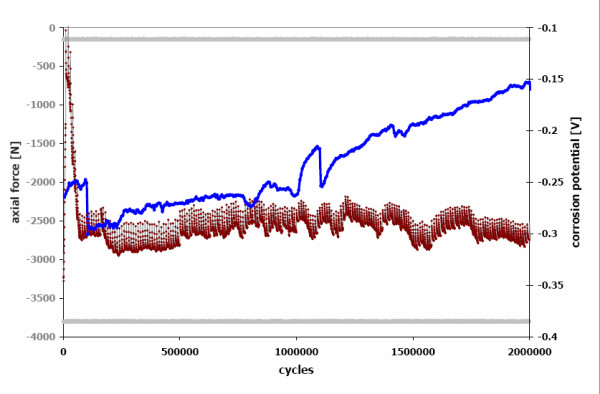
**Free redox potential with frequent repassivation processes in the particle-contaminated modular neck interface (red curve) compared with a clean joining (blue curve)**.

Contamination with cortical bone particles in the interface was found to be the main influence factor on the fatigue behaviour of the titanium alloy neck adapter. With a hip contact force of 3800 N the contaminated titanium alloy neck adapters failed after 3 million cycles (range 2.7 to 3.3) while no failure occurred under clean interface conditions (run-out limit 10 million cycles). This is mainly due to the increase of micromotions and the resulting fretting corrosion in the modular neck interface. In clean conditions, the load has to be increased up to 5300 N to provoke a similar failure mode. The redox potential measures fretting corrosion and repassivation on metallic surfaces. It shows impressively that introducing cortical particles into the modular neck interface generates a mechanical disturbance which triggers repassivation processes in high frequency (Figure 16).

## Discussion

This study confirmes that the failure of the adapter cannot be attributed to any material or processing deviation or incorrect dimensioning. A twisted assembling of the adapter can also be excluded.

Due to mechanical perturbation in the modular connection, micro-movements caused fretting on the surface. Microcracks developed in the fretting zone, ultimately leading to the dynamic fatigue fracture of the implant. In 87% of the cases, fretting was accompanied by corrosion [17,20-25] as it initiates crevice corrosion [26,27]. The combination of fretting and crevice corrosion destroys the passive layer permanently. The electrochemical reactions provoke a shift in the pH value into the acidic range. The corrosion process generates a fissured surface of the connecting cone and can also generate microcracks. The examinations revealed that corrosion does not trigger the implant failure but does accelerate the process [26]. Cone adapters free from corrosive attack last the longest period up to failure (Figure 10).

In general, the titanium alloy provides excellent corrosion resistance and high biocompatibility because it quickly develops a thin passive layer with a thickness under 1 μm. Under inadequate conditions of cone assembling and in case of micro-movements within the modular connection, the protective passive layer is abraded initiating a continued abrasion and repassivation process which depletes the oxygen inside the crevice [17]. The metallic surface in the crevice becomes anodic relative to the outer surface, thus triggering an electrolytic process. The presence of chloride ions which disturb the passivity, induces pitting corrosion in the crevice leading to a quick dissolution of the metal accompanied by an acidification of the electrolyte [12,20,21,28].

Inside the layer, the elements calcium and phosphor could be detected and verified by different analytical methods. The crystalline composition of the calcium phosphate compound seems to indicate that the wear debris contained bone particles. Impurities which get inside the cone during intraoperative assembly may contribute to a mechanical perturbation which manifests itself as micro-movements which initiates fretting.

The study suggests the following hypothesis as to the cause of the damage. Fretting occurs when two surfaces in contact experience small amplitude oscillary relative motion; damage is induced on the fretting region. If the fretting fatigue strength of the material is exceeded, microcracks develope on the surface. In addition, tribochemically activated particles can discharge their content from the surface. These particles react with oxygen spontaneously, thus leading to fretting corrosion.

The severity of corrosion depends mainly from the frequency of the fretting action. For lower cyclic rates such as in a hip implant, the debris is usually oxidized if the environment is chemically active. The repeated removal of oxide films due to continued abrasion and repassivation produces thick oxide layer's. Such oxides are normally harder than the virgin titanium alloy leading to greater surface damage [29-31] These damages then accelerate the crack nucleation [32,33].

Fretting reduces the fatigue strength of the titanium significantly [34]. To a great extent, this can be avoided by using of cobalt-chromium. The criteria listed in Table 3 show the comparison of the different materials which have been verified by biomechanical investigation. At a load of 5300 N the adapter made of the cobalt-based alloy did not break. At this high load level, the titanium adapter fractured after 2.45 million cycles.

**Table 3:** Assessment of the cone adapter made of the different materials - (ooo = excellent oo = good o = moderate)

	TiAl6V4	CoCr29Mo6
**mechanical properties**		

fatigue strength	ooo	ooo

stiffness/modulus of elasticity	oo	ooo

notch sensitivity	o	ooo

crack propagation	o	oo

abrasion	o	ooo

		

**corrosion characteristics**		

passive layer	ooo	ooo

re-passivation	ooo	oo

fretting corrosion	o	ooo

crevice corrosion	ooo	oo

allergic potential	ooo	o

The surface damage of the titanium alloy adapters caused by the microcracks or by corrosive deterioration accelerates the propagation of cracks by the cyclic loads bringing about the dynamic fatigue failure of the adapters. Micro-movements cause fretting in the cone connection. They can be increased by contamination of the cone connection through tissue or other particles intraoperatively. To anticipate this process any contamination of the connection should be avoided and the components dried before assembling. For this purpose abrasion-resistant cleaning rods are supplied together with the implants.

## Conclusions

The change of the material of the adapter from titanium alloy to a cobalt-based alloy (CoCr29Mo6) increases the safety of the cone connection significantly. The combination of the cobalt-based alloy and the titanium alloy of the shaft shows a considerably higher rigidity. The smaller micro-movements reduce abrasion. Furthermore, the highly stable passive layer of the cobalt-based alloy provides an improved resistance against fretting. Due to its structure, the cobalt alloy has a much lower notch sensitivity compared to the titanium alloy. This enhances fatigue strength.

Among patients treated with the titanium alloy neck adapter, a combination of different parameters was identified as risk factors of implant failure. The parameters are intraoperative particle contamination of the cone connection, excessive loading due to a patient weight above 100 kg or high activity level, and male gender. In addition, the risk for failure rises with CCD angles of the cone adapter of 135° and smaller.

## Competing interests

The authors TMG, TW, WB, HPK are all employees of Aesculap AG Tuttlingen, Germany

## Authors' contributions

The authors TMG, TW, WB, HPK have made substantial contributions to conception and design, or acquisition of data, or analysis and interpretation of data; 2) TMG, TW, WB, HPK have been involved in drafting the manuscript or revising it critically for important intellectual content; and 3) TMG, TW, WB, HPK have given final approval of the version to be published.

## Pre-publication history

The pre-publication history for this paper can be accessed here:

http://www.biomedcentral.com/1471-2474/11/3/prepub
